# Ficolin-2: A potential immune-related therapeutic target with low expression in liver cancer

**DOI:** 10.3389/fonc.2022.987481

**Published:** 2022-11-08

**Authors:** Li-ting Wang, Qiu-ling Zeng, Shao-lan Jiang, Zhen-yu Chen, Xiao-ling Wang, Ling Li, Xiaolong Li

**Affiliations:** ^1^ The First Clinical College of Guangxi Medical University, Nanning, China; ^2^ Department of Pathology, The People’s Hospital of Guangxi Zhuang Autonomous Region, Nanning, China; ^3^ Department of Cell Biology and Genetics, Key Laboratory of Longevity and Agingrelated Diseases of Chinese Ministry of Education, School of Pre-Clinical Medicine, Guangxi Medical University, Nanning, China

**Keywords:** ficolin-2 lectin, liver cancer, bioinformatics, immunohistochemical test, immune-related

## Abstract

**Objective:**

This study aimed to investigate the role of ficolin-2 (*FCN2*) in the development and course of hepatocellular carcinoma (HCC) and to contribute to the evolution of innovative HCC therapeutics.

**Methods:**

Oncomine, GEPIA (Gene Expression Profiling Interactive Analysis), TISIDB (Tumor Immune System Interactions and Drug Bank database), UALCAN (University of Alabama at Birmingham Cancer data analysis portal), UCSC (University of California, Santa Cruz), R package, the Kaplan–Meier technique, Cox regression analysis, LinkedOmics, Pearson’s correlation, and a nomogram were used to investigate the prognostic value of *FCN2* in HCC. Co-expressed genes were screened. A protein–protein interaction network was created using the STRING database. Finally, immunohistochemistry was performed to establish the expression of *FCN2* in HCC tissues. A pan-cancer study centered on HCC-related molecular analysis was also conducted to look for a link between *FCN2* and immune infiltration, immune modulators, and chemokine receptors.

**Results:**

In HCC tissues, the expression of *FCN2* was observed to be lower than that in normal tissues. This was connected to the HCC marker alpha-fetoprotein, showing that *FCN2* is involved in the development and progression of cancer. *FCN2* may act through *Staphylococcus aureus* infection, lectins, and other pathways. Furthermore, at the immune level, the expression of *FCN2* in HCC was associated with some immune cell infiltration, immunomodulators, and chemokine receptors.

**Conclusion:**

*FCN2* may be an immune checkpoint inhibitor for HCC, creating a breakthrough in the treatment of HCC.

## Introduction

Liver cancer is a deadly tumor with a high fatality rate that is getting worse year after year (1). Serological tests for alpha-fetoprotein (AFP), alkaline phosphatase (ALP), alanine transferase (ALT), total bilirubin (T-BIL), total protein (TP), albumin (ALB), and hepatitis B surface antigen (HBsAg) are crucial for the diagnosis of liver cancer. However, the serum AFP test is the most sensitive method (2). The aberrant expression of AFP in liver cancer is a crucial determinant in clinical diagnosis and liver cancer prediction (3). In the clinical application of hepatocellular carcinoma (HCC), traditional diagnostic methods such as serum AFP have limited specificity and sensitivity (4), and current data show that there is no single biomarker for the diagnosis of HCC, especially in the inchoate stage of development (5). Therefore, specific biomarkers are expected to improve the prognosis of patients by improving the diagnosis of HCC, especially the early diagnosis of tumors (5).

One of these biomarkers is synthesized and discharged by hepatocytes within the human body. Ficolin-2 (*FCN2*) is an innate immune pattern recognition molecule that motivates the complement cascade and opsonizes miscellaneous pathogens (6). Transforming growth factor beta (TGF-β) helps *FCN2* participate in the metastasis of HCC and in epithelial-to-mesenchymal transition (EMT). Low *FCN2* levels have been associated with the aggressive metastatic features of HCC and therefore could be used as a predictive indicator for the disease-free survival (DFS) of HCC patients (7). Based on this assumption, we believe that *FCN2* could be used as a biomarker in the early stages of liver cancer, but more research into the subject is required.

Previous studies have mostly focused on the aberrant expression of *FCN2* in liver cancer (8). In this study, we not only provided a more comprehensive bioinformatics analysis but also obtained support from clinical experimental results. We started by screening genes using a network database, then performed prognostic analysis on the screened genes, and finally obtained the *FCN2* gene required. Subsequently, a series of analyses were carried out on *FCN2*, such as pan-cancer analysis, differential expression analysis, clinicopathological analysis, and immune infiltration analysis. Finally, immunohistochemical samples obtained from the clinic were analyzed. Combining these methods, we have concluded that *FCN2* could be an immune checkpoint inhibitor for liver cancer, which we hope will provide a breakthrough point in the cure of liver cancer.

## Methods

### Oncomine

The expression of *FCN2* messenger RNA (mRNA) in liver cancer was investigated using the Oncomine 4.5 database (esophageal carcinoma, ESCA). Oncomine.org is the world’s largest oncogene chip database and is a data mining platform in general. A number of ESCA studies involving Cuichard liver cell carcinoma, Roessler liver cancer, and The Cancer Genome Atlas (TCGA) liver cancer research were dissected and data obtained. The expression of *FCN2* in HCC and in normal tissues was then investigated. A related inquiry was carried out based on the comparison of the transcription results. The common values and multiples of *p*-values were as below: *p* < 0.01; multiple of difference, 1.5; gene arrangement, 10%; data type, mRNA.

### GEPIA

The recently established interactive web server Gene Expression Profiling Interactive Analysis (GEPIA) (9, 10), which examines tumor and natural swatch sequencing expression data, was used to analyze the expression of *FCN2* in liver cancer and normal liver tissues. GEPIA was also utilized to obtain the mRNA expression data of *FCN2* in the liver cancer tissues in this study. A network analysis was then implemented. Gene expression profiles were obtained from 369 liver cancers and 50 normal cases.

### Genome browser database at the University of California, Santa Cruz

The UCSC (University of California, Santa Cruz) database (https://genome.edu/) is one of the most widely used in biology. We used this database to download a single standardized global cancer dataset, and then retrieved and screened the gene expression data of *FCN2* in each sample. To determine the expression differences between normal and tumor samples, R software (version 3.6.4) was used for analysis according to previous studies. The significance of the differences was determined using the unpaired Wilcoxon rank-sum and signed-rank tests. The gene expression data of *FCN2* in each sample, as well as the gene sequences retrieved, were examined using the embryonic stem cell-specific (ECSC) dataset.

### TISIDB

TISIDB (Tumor Immune System Interactions and Drug Bank database; http://cis.hku.hk/TISIDB/) is a tumor and immunity-related database that collects a variety of data. It can carry out a large number of special screenings of genes and report genes related to T-cell-mediated killing or immunotherapy. It also includes data from TCGA on the links between genes and their immunological functions in 30 different tumor types. The TISIDB database was used to look for a link between *FCN2* expression and the liver cancer stage.

### UALCAN data analysis

UALCAN (University of Alabama at Birmingham Cancer data analysis portal) (11, 12) draws on the TCGA database and employs the capacities of cancer data mining and online examination to analyze and handle data effectively. Besides identifying relevant genetic biomarkers, it also includes expression profile and survival analyses and can directly query related data in other databases using relevant links. In summary, this website can be used to efficiently mine and analyze TCGA data with simple operations. UALCAN was utilized to analyze the expression of *FCN2* in normal and liver cancer tissues in this study.

### Cancer Cell Line Encyclopedia

The Cancer Cell Line Encyclopedia (CCLE; https://sites.broadinstitute.org/ccle/) is a database of cancer cell lines maintained by the Broad Institute that includes relevant data on previous cell lines. By searching for cell lines, annotations, and genes on this website, the expression level of the specified gene in each tumor cell line can be determined.

### cBioPortal

The cBioPortal (http://www.cbioportal.org) currently contains 225 cancer studies. Changes in the levels of *FCN2* in the HCC samples from TCGA were analyzed using cBioPortal. The search parameters included mutations and mRNA expression. The OncoPrint tab displays the genetic mutation profile of each sample. Furthermore, the network shows the biological interaction network of *FCN2* from the common pathway database using color coding and screening options grounded on the periodicity of each gene’s genome change, including neighboring genes that change more frequently. Kaplan–Meier plots were drawn to show that *FCN2* gene mutations are associated with the overall survival (OS) of patients with HCC. A logarithmic rank test was performed to explain the survival curve.

### Human Protein Atlas

Proteomics, transcriptomics, and systems biology data are used to map tissues, cells, and organs in the Human Protein Atlas (HPA) database (https://www.proteinatlas.org). This database can be used to assess the protein expression in tumor and normal tissues, as well as the OS of patients with tumors. We used the HPA database to obtain the immunochemistry data for related genes.

### Search Tool for the Retrieval of Interacting Genes/Proteins

The Search Tool for the Retrieval of Interacting Genes/Proteins (STRING) database (https://www.string-db.org/) is an online database that investigates protein interactions that have already occurred. This website can assist in identifying key regulatory genes. This website’s supremacy stems from the fact that it contains the most species and interaction data. We created a protein–protein interaction (PPI) network for *FCN2* using the STRING database. From the PPI network diagram, we found 10 fCN2-related genes. Furthermore, analyses using Gene Ontology (GO) and Kyoto Encyclopedia of Genes and Genomes (KEGG) pathways were conducted for *FCN2* and the 10 genes discovered.

### Immunohistochemistry

The expression of *FCN2* was examined using immunohistochemistry. Samples were collected from 30 patients with liver cancer at the Guangxi Zhuang Autonomous Region’s People’s Hospital. The samples were paraffin-fixed, cut into serial sections, and incubated with rabbit ficolin-2/ficolin-B polyclonal antibody (bs-13162R; Bioss, Woburn, MA, USA) at 4°C overnight. The sections were then dyed with hematoxylin and eosin (HE). Every procedure was executed as per the instructions in the SP kit. The number of positively stained cells during immunohistochemical labeling was determined using the antigen content, distribution density, tag method, and susceptibility. The distribution density of the positive results increased with the increase of antibody content, and the color development was brighter. Blue was the negative marker, light yellow indicated a slightly positive mark, brownish yellow was the moderately positive marker, and dark brown was the highly positive marker. In general, the more strongly positive regions should be photographed.

We took several representative images using the OlymbusX21 microscope. ImageJ was then used to execute a general morphometric analysis of each image. The photic density and positive area data for normal and cancer tissues were procured by measuring the chosen dyeing region using the ImageJ parameter. The higher the optical density and the more positive the expression, the higher the average level. Finally, statistical methods were used to determine whether there was a difference in the *FCN2* expression between the normal and cancer groups.

### Kaplan–Meier plotter

The Kaplan–Meier plotter (13) is capable of assessing the correlation between the expression of all genes (mRNA, miRNA, and protein) and survival in more than 30,000 samples from 21 tumor types, including breast, ovarian, lung, and gastric cancer. Sources for the databases include the Gene Expression Omnibus (GEO), the European Genome–Phenome Archive (EGA), and TCGA. The primary purpose of the tool is the meta-analysis-based discovery and validation of survival biomarkers.

### Statistical analysis

The Wilcoxon rank-sum test was used because normality monitoring made it clear that the specimen failed (*p* < 0.05). According to the Wilcoxon rank-sum test, the tumor was lower than normal, with a median difference of −0.084 between the two groups (−0.11 to 0.054). The diversity (*p* < 0.001) was statistically significant. Statistical analysis was executed with the R program (primarily ggplot2), with the following levels of significance: ns, *p* < 0.05, *p < 0.05, **p < 0.01, and ***p < 0.001.

## Result

### Screening datasets yielded differentially expressed genes

**Figure 1:**
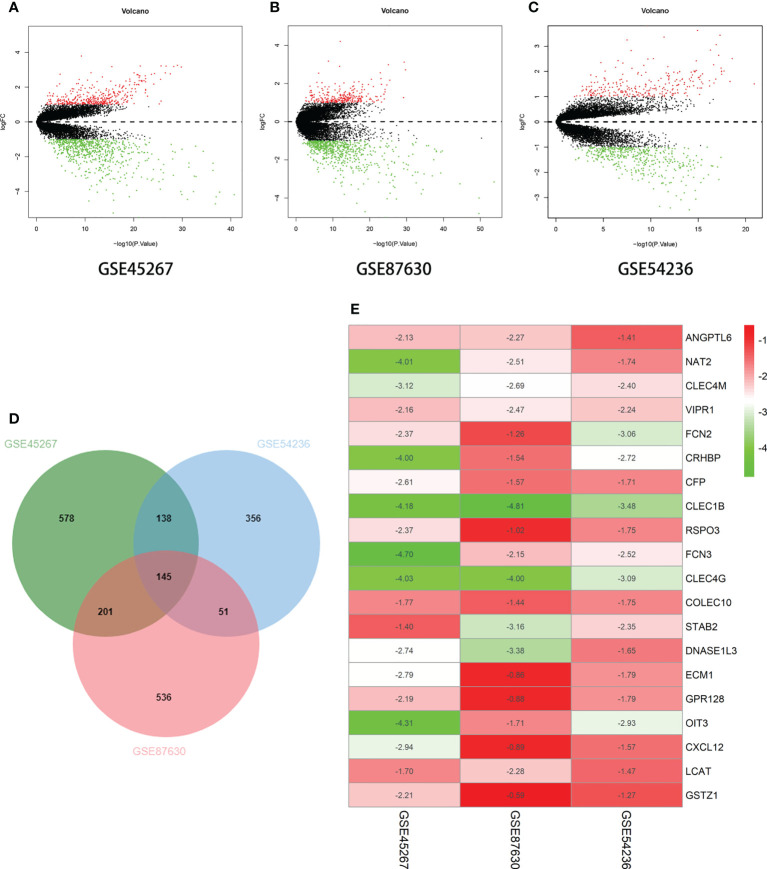
Differential expression of multiple genes in the three datasets. **(A)** GSE45267 dataset. **(B)** GSE87630 dataset. **(C)** GSE54236 dataset. **(D)** Identification of the significantly differentially expressed genes (DEGs) in hepatocellular carcinoma (HCC) in the GSE45267, GSE87630, and GSE54236 datasets established the intersection of the DEGs. **(E)** The 20 genes with significant downregulation obtained using the RobustRankAggreg (RRA) algorithm. Taking |logFc| > 1 as the boundary value, *p* < 0.05.

In this study, we wanted to determine whether there were any differences in the gene expression between liver cancer and robust liver tissues. The GSE45267, GSE87630, and GSE54236 datasets from the National Center for Biotechnology Information (NCBI) were chosen as being related to liver cancer. Differentially expressed genes (DEGs) were filtered out using bioinformatics and R analysis, and the RobustRankAggreg (RRA) algorithm was employed to determine 20 genes with unique downregulated expressions. In this study, the 20 downregulated genes were selected for further analysis (**Figure 1**). It was discovered that liver cancer tissues had lower levels of *ANGPTL6*, *CFP*, *CLEC1B*, *CLEC4G*, *CLEC4M*, *COLEC10*, *CRHBP*, *CXCL12*, *DNASE1L3*, *FCN2*, *FCN3*, *GSTZ1*, *LCAT*, *NAT2*, *OIT3*, *RSPO3*, *VIPR1*, *STAB2*, *ECM1*, and *GPR128* expression compared to normal liver cells.

### Expression levels of screened genes in liver cancer and normal liver tissues

**Figure 2:**
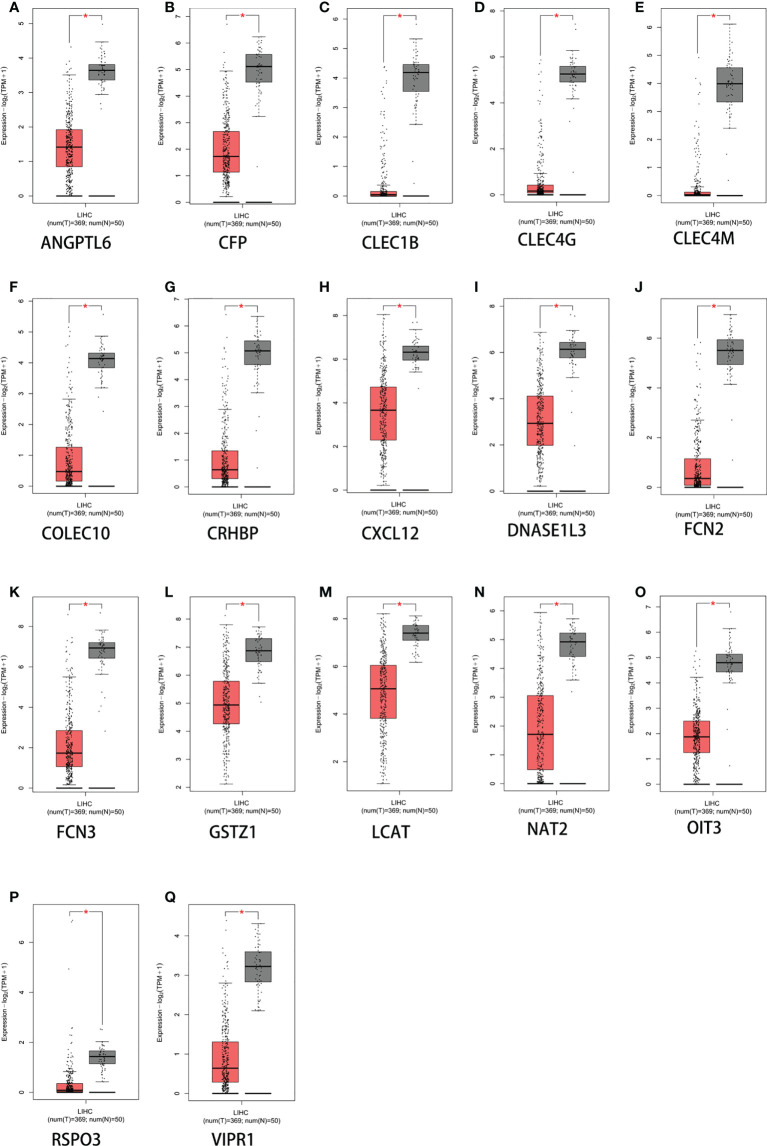
Expression levels of 17 genes based on GEPIA2 (Gene Expression Profiling Interactive Analysis web server). **(A)** *ANGPTL6*. **(B)** *CFP*. **(C)** *CLEC1B*. **(D)** *CLEC4G*. **(E)** *CLEC4M*. **(F)** *COLEC10*. **(G)** *CRHBP*. **(H)** *CXCL12*. **(I)** *DNASE1L3*. **(J)** *FCN2*. **(K)** *FCN3*. **(L)** *GSTZ1*. **(M)** *LCAT*. **(N)** *NAT2*. **(O)** *OIT3*. **(P)** *RSPO3*. **(Q)** *VIPR1*. *p  < 0.05. The *y*-axis represents the relative log2 expression value (TPM + 1).

The 17 extracted genes (*ANGPTL6*, *CFP*, *CLEC1B*, *CLEC4G*, *CLEC4M*, *COLEC10*, *CRHBP*, *CXCL12*, *DNASE1L3*, *FCN2*, *FCN3*, *GSTZ1*, *LCAT*, *NAT2*, *OIT3*, *RSPO3*, and *VIPR1*) were validated using the GEPIA2 (Gene Expression Profiling Interactive Analysis) database. *STAB2* was not found, and the levels of *GPR128* and *ECM1* were not significant. In contrast to their expression in normal liver tissues, the retrieved genes were only slightly expressed in HCC tissue samples (**Figure 2**). This indicates that both the GEO and the TCGA database have confirmed that these genes are indeed significantly underexpressed in liver cancer.

### Assessment of the prognostic value of the screened genes in liver cancer and determination of the target gene *FCN2*

**Figure 3:**
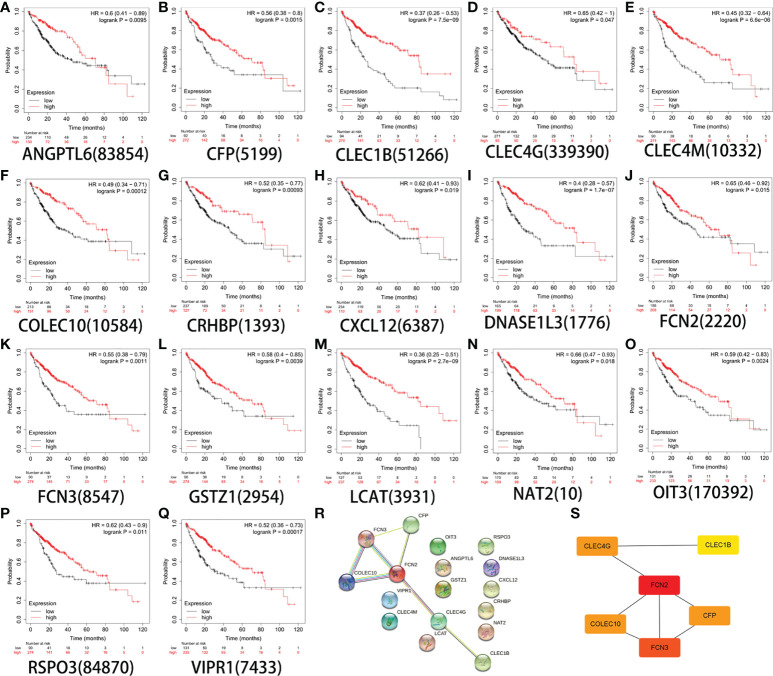
Survival analysis of 17 genes. **(A)** *ANGPTL6*. **(B)** *CFP*. **(C)** *CLEC1B*. **(D)** *CLEC4G*. **(E)** *CLEC4M*. **(F)** *COLEC10*. **(G)** *CRHBP*. **(H)** *CXCL12*. **(I)** *DNASE1L3*. **(J)** *FCN2*. **(K)** *FCN3*. **(L)** *GSTZ1*. **(M)** *LCAT*. **(N)** *NAT2*. **(O)** *OIT3*. **(P)** *RSPO3*. **(Q)** *VIPR1*. **(R)** Constructed protein–protein interaction (PPI) network of the important differentially expressed genes (DEGs) using STRING. **(S)** Use of the Cytoscape plug-in MCODE to select the most important module from the PPI network.

The Kaplan–Meier plotter database was used to determine and evaluate the prognostic value of 20 genes (*p* < 0.05). To ensure the simplicity of the experiment, genes that showed no statistical significance were eliminated. Finally, the genes *ANGPTL6*, *CFP*, *CLEC1B*, *CLEC4G*, *CLEC4M*, *COLEC10*, *CRHBP*, *CXCL12*, *DNASE1L3*, *FCN2*, *FCN3*, *GSTZ1*, *LCAT*, *NAT2*, *OIT3*, *RSPO3*, and *VIPR1* were obtained (**Figures 3A–Q**). The PPI network of DEGs was affected and was analyzed using the STRING database (**Figure 3R**). Using the cell type Minimal Common Oncology Data Elements (mCODE), we identified the most important nodes and retrieved six central nodes (**Figure 3S**). Among them, *FCN2* was a central node.

### Expression of *FCN2* in pan-cancer

**Figure 4:**
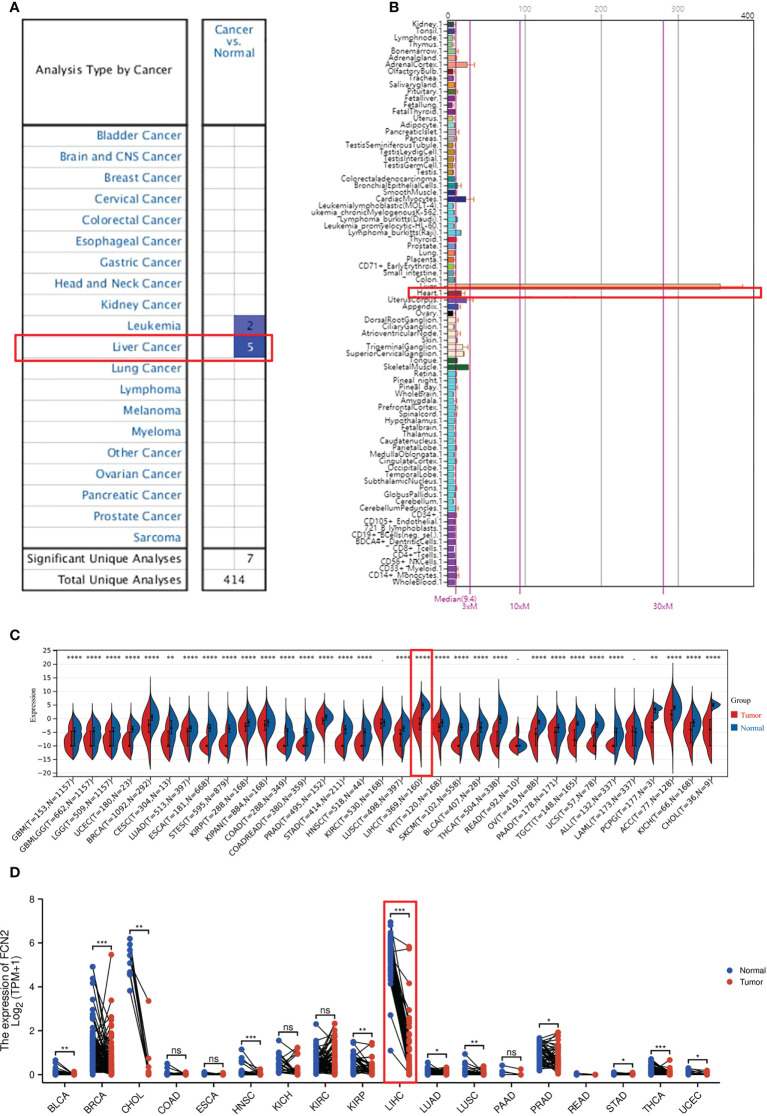
Pan-tissue expression of *FCN2*. **(A)** Pan-cancer expression of *FCN2* in the Oncomine database. **(B)** Expression of *FCN2* in normal tissues in the BioGPS database. **(C)** Pan-cancer expression of *FCN2* in the UCSC (University of California, Santa Cruz) database. **(D)** Log2 transformation of the pan-cancer expression value of *FCN2* in the UCSC database. *p < 0.05, **p < 0.01, ***p < 0.001, ****p < 0.0001, ns: not statistically significant.

The expression of *FCN2* mRNA in various tumor tissues was investigated. The Oncomine database was used to examine the expression of *FCN2* mRNA in various cancers and normal clinical samples (**Figure 4A**). A total of 639 datasets with a total of 78,661 samples were selected. The results showed that, in contrast to normal tissues, the mRNA expression of *FCN2* is downregulated in leukemia and liver cancer, demonstrating that the transcription of *FCN2* is tumor-specific.

The expression data of *FCN2* in pan-cancer can be procured from the BioGPS database (**Figure 4B**). The expression data of the *FCN2* gene in each sample (**Figure 4C**) were derived from a unified and standardized dataset from the UCSC. We also screened the following tissue sources: protopathic solid tumors, protopathic tumors, normal tissue, protopathic blood-derived cancer—bone marrow, and protopathic blood-derived cancer—peripheral blood samples. We then utilized R software to calculate the expression differences between normal and tumor tissues in each tumor (version 3.6.4). It was discovered that the expression levels in 31 malignancies were significantly downregulated, including glioblastoma (GBM), breast cancer (BRCA), cervical squamous cell carcinoma and endocervical adenocarcinoma (CESC), lung adenocarcinoma (LUAD), kidney renal papillary cell carcinoma (KIRP), colon adenocarcinoma (COAD), prostate adenocarcinoma (PRAD), liver hepatocellular carcinoma (LIHC), tenosynovial giant cell tumors (TGCT), and adenoid cystic carcinoma (ACC), utilizing unopposed Wilcoxon rank-sum and signed-rank monitoring. A log2(*x* + 0.001) transformation was also applied for each expression value (**Figure 4D**), and the statistical significance was determined using paired Wilcoxon signed-rank test.

### Transcription levels of *FCN2* in liver cancer and normal tissues

**Figure 5:**
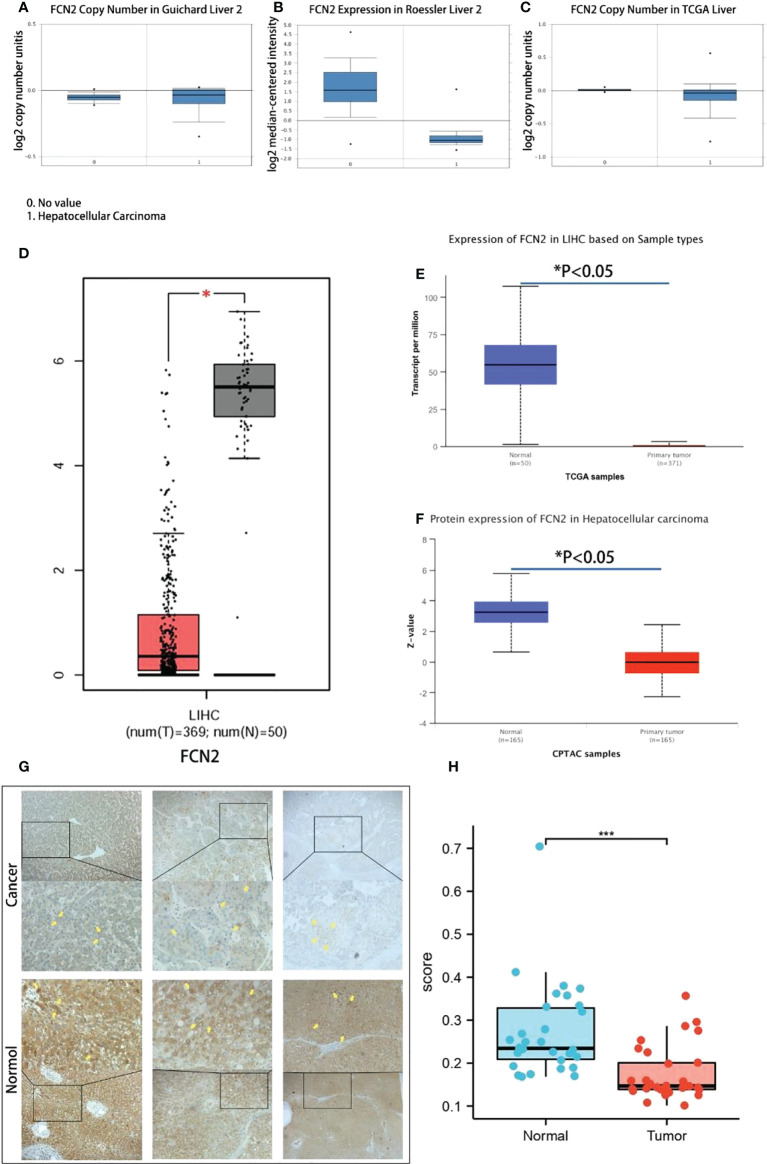
Transcription levels of *FCN2* in liver cancer and normal tissues. **(A–C)** Expression differences of the *FCN2* gene between liver cancer tissues and normal tissues in the Oncomine database. **(D)** *FCN2* mRNA expression in liver cancer tissue and normal tissue based on the GEPIA2 database (TCGA tumors *vs*. TCGA normal). **(E, F)** *FCN2* protein expression in hepatocellular carcinoma in the UALCAN database. **(G)** Part of the results of the immunohistochemistry experiments. **(H)** Average optical density of liver cancer tissues and adjacent tissues from 30 liver cancer patients. *TCGA*, The Cancer Genome Atlas. *p < 0.05, ***p < 0.001.

An Oncomine database search of the expression of *FCN2* in diverse liver cancer studies produced three studies showing a lower expression of *FCN2* in LIHC tissues compared to normal liver tissues (*p* = 1E−4, multiple of difference: 2) (**Figures 5A–C**).

The transcription and translation results of *FCN2* mRNA in 369 liver cancer and 50 normal liver tissues were acquired using the GEPIA2 database. The findings revealed that the mRNA and protein expressions of *FCN2* were lower in liver cancer tissues than those in normal liver tissues in all cases (*p* < 0.05) (**Figure 5D**). To confirm these results in depth, we used the UALCAN online analysis tool to further analyze the *FCN2* gene, the result of which was similar in that the expression level of *FCN2* in HCC cells was markedly lessened compared to that in normal cells (**Figures 5E, F**).

Liver cancer samples from 30 patients were taken from the People’s Hospital of Guangxi Zhuang Autonomous Region and immunohistochemistry staining was performed. Each staining procedure followed a rigorous set of guidelines. Five fields of view were randomly chosen from each sample after staining, and the average optical density value was determined using ImageJ. SPSS 19.0 was used for statistical analysis (IBM Inc., Armonk, NY, USA).

The areas of positive staining were brown, and the microscopy results revealed that normal liver tissues had bigger areas of positive staining than did liver cancer tissues (**Figure 5G**). Normal liver tissues had an average positive area of 1,783,320.5318 m^2^ and an average optical density of 0.25662176264, while tumor tissues had an average positive area of 1,357,014.3320 m^2^ and an average optical density of 0.16741577631. The normal and tumor groups had significance values of 0.200 and 0.086, respectively, suggesting that the positive staining area followed a normal distribution. Analysis of the *t*-test results revealed a significance of 0.036 (0.05), suggesting that the two sets of data are quite dissimilar and that the positive staining zone of the cancer group is smaller than that of the normal group (**Figure 5H**). The expression of *FCN2* mRNA in liver tumor cell lines was shown to be at a low level using RNA sequencing (RNAseq) (**Attachment 1**). Affymetrix analysis exhibited the expression of *FCN2* mRNA in all tumor cell lines to be above 3.2. These studies have confirmed the significantly low expression of *FCN2* in liver cancer tissues from different databases, as well as mRNA, protein, and clinical specimens.

### Association of *FCN2* expression and clinicopathological variables

As shown in (**Attachment 2**), the TCGA dataset yielded 1,374 clinical and gene expression data on primary cancers. The sample included data from 253 men and 121 women, with a median age of 61 years. The protein expression of *FCN2* was found to be strongly associated with height (*p* = 0.024) and the AFP level (*p* = 0.004) in the correlation analysis.Other clinicopathological features showed no association with *FCN2* expression. The downregulation of *FCN2* in HCC was linked to height (*p* = 0.018) and the AFP level (*p* = 0.003) in the univariate analysis (**Attachment 3**).

**Figure 6:**
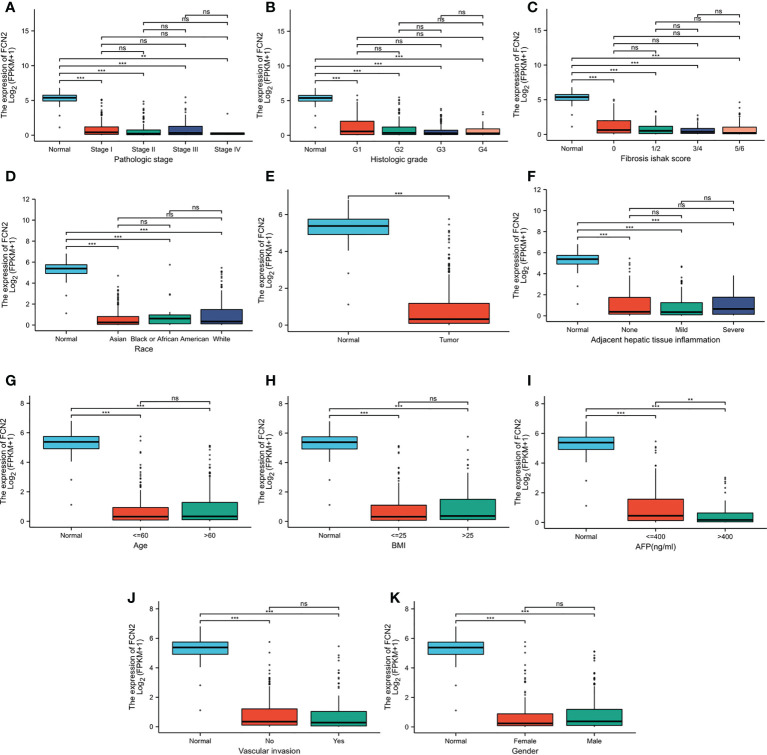
Different expression levels of *FCN2* in liver hepatocellular carcinoma (LIHC) based on The Cancer Genome Atlas (TCGA) analyzed using R. **(A)** Pathologic stages. **(B)** Histological grade. **(C)** Fibrosis Ishak scale scores. **(D)** Race. **(E)** Normal tissue and liver cancer. **(F)** Adjacent hepatic tissue inflammation. **(G)** Age **(H)**. BMI. **(I)** Alpha-fetoprotein (AFP). **(J)** Vascular invasion. **(K)** Gender. *p < 0.05, **p < 0.01, ***p < 0.001. ns: not statistically significant.

To further verify the association between *FCN2* and liver cancer, we analyzed the expression of the *FCN2* gene in several markers using the R tool. The expression levels of *FCN2* varied between the low- and high-AFP groups (*p* < 0.001) (**Figures 6A–K**). This indicates that *FCN2* has certain clinical importance, whether from the high and low *FCN2* expression groups (median) or the expression analysis of normal liver tissue and different liver cancer subgroups, and could be a complementary gene for AFP detection in liver cancer.

### Clinical value of *FCN2* in prognosis

**Figure 7:**
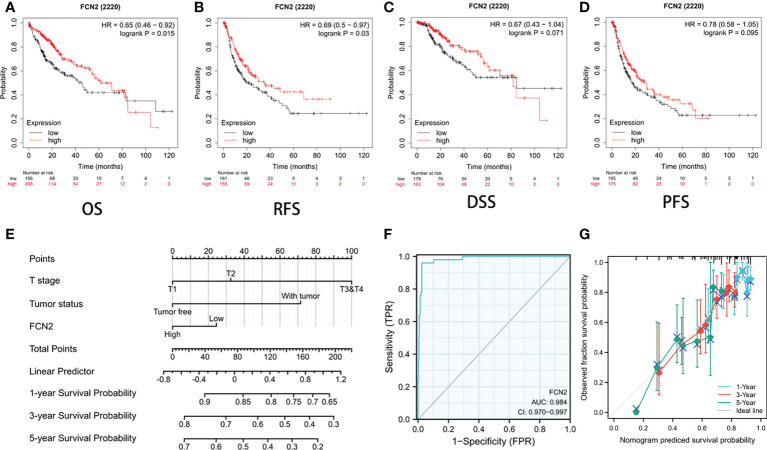
Prognostic value of *FCN2* in liver cancer. **(A–D)** Overall survival (OS) **(A)**, relapse-free survival (RFS) **(B)**, disease-specific survival (DSS) **(C)**, and patient-free survival/progression-free survival (PFS) **(D)**. **(E)** Nomogram predicting the 1-, 3-, and 5-year OS probability. **(F)** C-index of the prognostic model nomogram for predicting the 1-, 3-, and 5-year OS probability in hepatocellular carcinoma (HCC) patients. **(G)** Prediction of the 1-, 3-, and 5-year OS probability using nomogram calibration plots.

The survival rates of the high and low *FCN2* expression level groups were compared to establish the predictive value of the expression of *FCN2* in HCC. According to the Kaplan–Meier survival analysis, the OS of patients with HCC with a low *FCN2* expression was poor [HR = 2.26 (1.58–3.24), *p* = 0.015], as was the DFS, compared to patients with a high *FCN2* expression [HR = 0.69 (0.5–0.97), *p* = 0.03] (**Figures 7A–D**).

The nomogram included data on the tumor status, T stage, and *FCN2* expression and was based on a Cox proportional hazards regression model (**Figure 7E**). The C-index of the prognostic model (**Figure 7F**) was 0.984 (95% CI = 0.970–0.997). We built a calibration plot to examine the similarity between the OS predicted by the prognostic model and the actual OS. The findings revealed that the nomogram’s prediction results were accurate (**Figure 7G**). These results imply that *FCN2* could be a useful prognostic indicator for HCC.

### Protein–protein interaction network analysis and enrichment analysis

**Figure 8:**
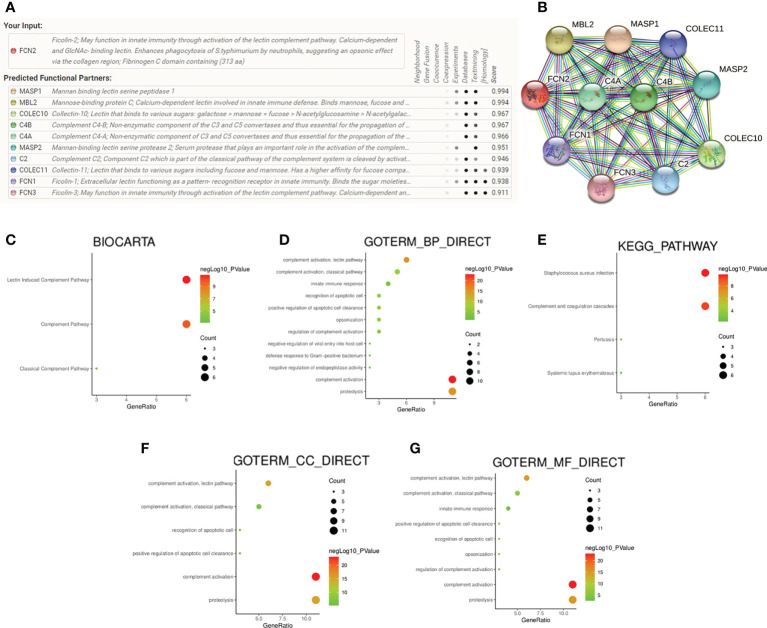
Protein–protein interaction network analysis and enrichment analysis. **(A, B)** Gene network diagram of interaction with *FCN2* created using the STRING database. **(C–G)** Diagrams of the analyses of the Kyoto Encyclopedia of Genes and Genomes (KEGG) and Gene Ontology (GO) pathways.

STRING was used to complete an online analysis of the FCN2 protein, producing a network diagram of the PPI between 10 genes and the *FCN2* gene (**Figures 8A, B**). The GO and KEGG pathway enrichment analyses for *FCN2* and the other 10 genes (**Figures 8C–G**) showed that *FCN2* is primarily involved in body immune processes, such as detecting and eliminating the molecular function of attaching to cellular constituents such as collagen trimer, mannose, complement, and opsonin and apoptotic cells, complement activation, and the lectin pathway. Signal pathway findings suggested that the *FCN2* gene participates in *S. aureus* infection, the coagulation cascade, and the lectin pathway of complement activation.

### Relationship between *FCN2* expression and hepatocellular carcinoma immune cell immersion

**Figure 9:**
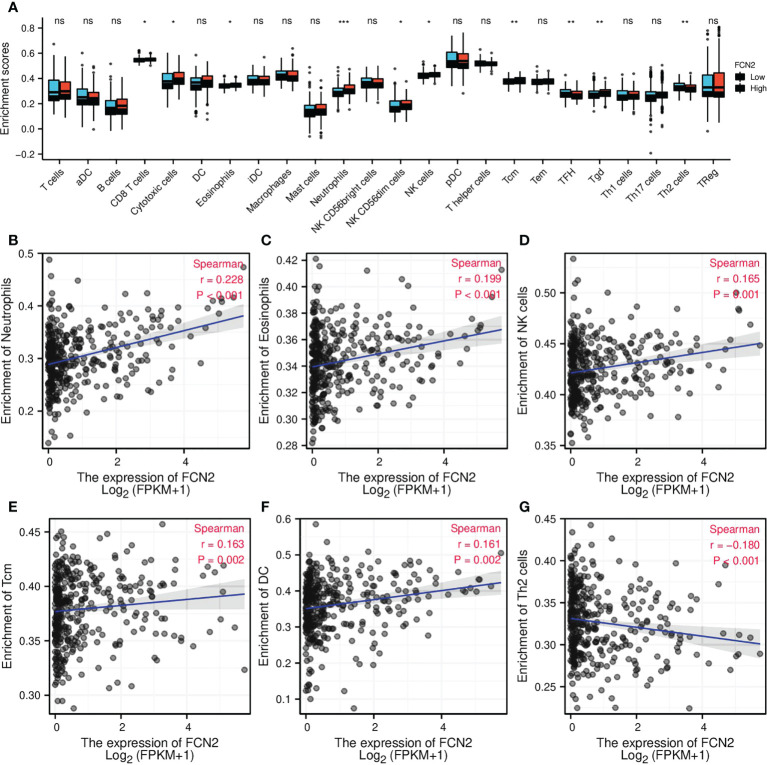
Correlation of *FCN2* expression with immune characteristics. **(A)** Differential distribution of the immune cells in patients with high and low *FCN2* expressions. **(B–G)** Correlation between the expression level of FCN2 and immune infiltration in hepatocellular carcinoma: **(B)** Neutrophils, **(C)** Eosinophils, **(D)** NK cells, **(E)** Tcm, **(F)** DC, **(G)** Th2 cells. *p < 0.05, **p < 0.01, ***p < 0.001. ns, no significance.

To explore whether there is a link between *FCN2* expression and tumor immune response, we conducted a single-sample gene set enrichment analysis (ssGSEA) to evaluate the immune cell infiltration in HCC tissues with various *FCN2* expression levels. According to the findings, the infiltration levels of T follicular helper (TFH) and T helper 2 (Th2) cells were considerably lower in patients with HCC who had low *FCN2* expression than those in patients who had high *FCN2* expression. Neutrophil infiltration was higher in patients with HCC who had low *FCN2* expression than in those who had high *FCN2* expression. The infiltration levels of T cells, activated dendritic cells (aDCs), B cells, dendritic cells (DCs), interstitial dendritic cells (iDCs), macrophages, mast cells, CD56^dim^ natural killer (NK) cells, plasmacytoid dendritic (pDC) cells, T helper cells, effector memory T (Tem) cells, Th1 cells, Th17 cells, and regulatory T cells (Tregs) in patients with high and low *FCN2* expressions were not substantially different (**Figure 9A**). We then examined the relationship between *FCN2* expression levels and immune cell infiltration in HCC and discovered that the expression of *FCN2* was positively linked to the number of neutrophils invading the tumor (*r* = 0.228, *p* < 0.001), eosinophils (*r* = 0.199, *p* < 0.001), NK cells (*r* = 0.165, *p* < 0.001), central memory T (Tcm) cells (*r* = 0.163, *p* < 0.001), and DCs (*r* = 0.161, *p* < 0.001) (**Figures 9B–F**, respectively). The number of Th2 cells entering the body showed a negative correlation (*r* = −0.180, *p* < 0.001) (**Figure 9G**).

We used the GEPIA database to investigate the relationship between *FCN2* expression and immune cell biomarkers in HCC in order to learn more about the role of *FCN2* in tumor immunity. As seen in **Table 1**, the expression of *FCN2* was negatively correlated with the M1 macrophage biomarker (*IRF5*), neutrophil biomarker (*ITGAM*), and DC biomarker (*ITGAX*) in liver cancer. The B-cell biomarker (*CD79A*), CD8^+^ T-cell biomarker (*CD8A*), CD4^+^ T-cell biomarker (*CD4*), M1 macrophage biomarker (*PTGS2*), M2 macrophage biomarkers (*CD163*, *VSIG4*, and *MS4A4A*), neutrophil biomarker (*CEACAM8*), and DC biomarker (*CD1C*) were positively correlated with *FCN2* expression. The above findings contribute to a better understanding of the link between *FCN2* and immune cell infiltration.

**Table 1:** Correlation analysis between *FCN2* and the biomarkers of immune cells in hepatocellular carcinoma (HCC) determined using the GEPIA database (Spearman’s correlation coefficient).

Immune cells	Biomarkers	*r*	*p*-value
B cells	*CD19*	−0.0065	0.89
	*CD79A*	0.13^a^	0.0075**^a^
CD8^+^ T cells	*CD8A*	0.11^a^	0.027*^a^
	*CD8B*	0.059	0.23
CD4^+^ T cells	*CD4*	0.34^a^	8.6E−13***^a^
M1 macrophages	*NOS2*	−0.07	0.16
	*IRF5*	−0.26^a^	6.5E−08***^a^
	*PTGS2*	0.35^a^	1.4E−13***^a^
M2 macrophages	*CD163*	0.29^a^	8.8E−10***^a^
	*VSIG4*	0.3^a^	2.6E−10***^a^
	*MS4A4A*	0.14^a^	0.0048**^a^
Neutrophil	*CEACAM8*	0.11^a^	0.031*^a^
	*ITGAM*	−0.097^a^	0.046*^a^
	*CCR7*	0.067	0.17
Dendritic cells	*HLA-DPB1*	0.063	0.2
	*HLA-DQB1*	−0.092	0.061
	*HLA-DRA*	0.051	0.29
	*HLA-DPA1*	0.079	0.1
	*CD1C*	0.13^a^	0.0087**^a^
	*NRP1*	−0.073	0.14
	*ITGAX*	−0.13^a^	0.0087**^a^

*p < 0.05; **p < 0.01; ***p < 0.001.

a Statistically significant results.

### Immunomodulators, chemokines, and receptors associated with *FCN2*

To confirm the role of *FCN2* in regulating LIHC immunity, we analyzed the correlation between *FCN2* and the tumor microenvironment (TME) immunological signatures.

**Figure 10:**
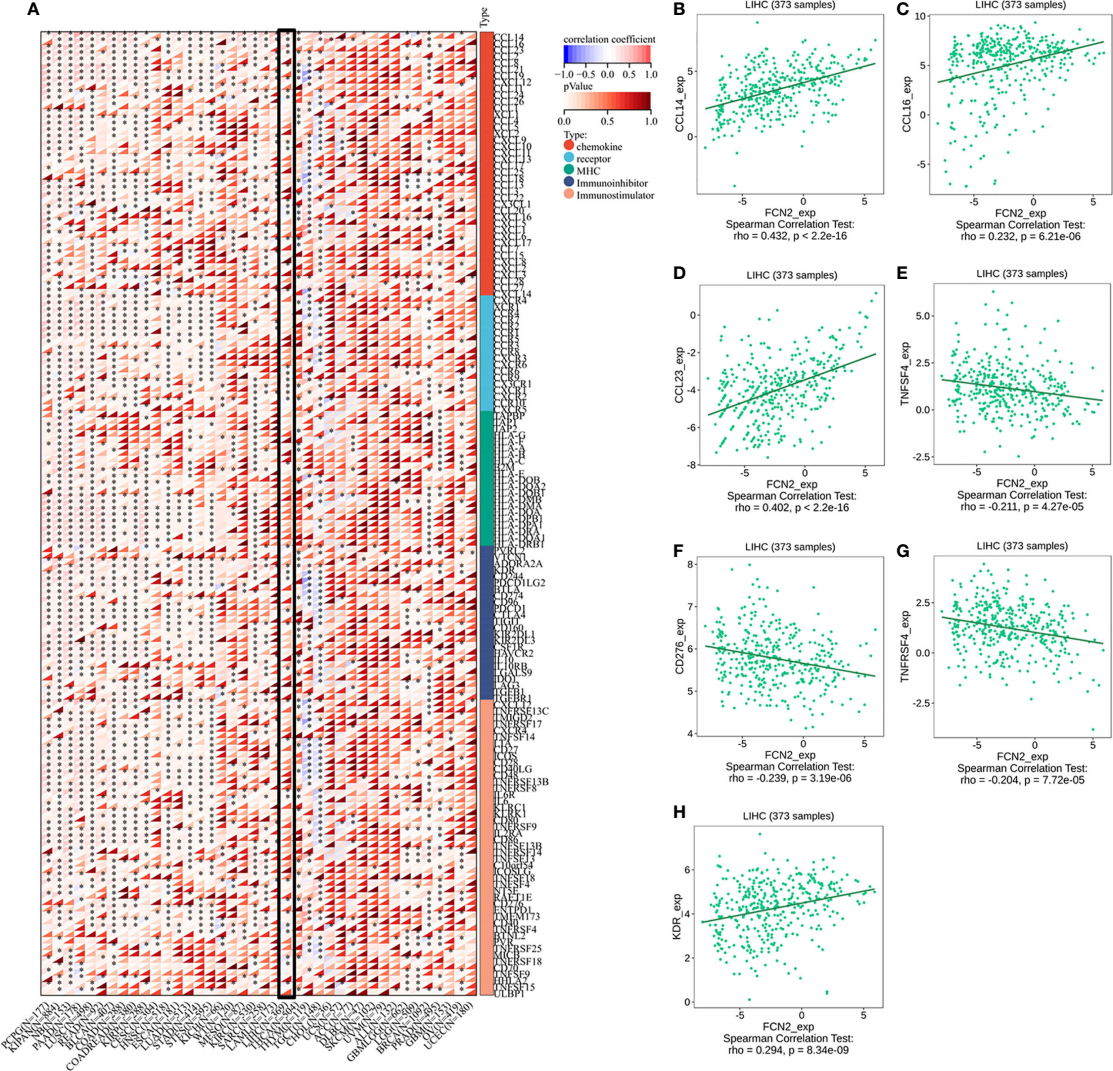
Immunomodulators, chemokines, and receptors associated with *FCN2*. **(A)** Distribution of the *FCN2* immunological scores in tumor and normal tissues.The *ordinate* reflects the distribution of the immunological scores in distinct groups, whereas the *abscissa* indicates the immune cell types. The Wilcoxon test was used to compare statistical differences between the two groups, and the Kruskal–Wallis test was used to determine the significance of the differences between three groups. (*a*) Heatmap of the immune cell scores. *Different hues* represent the varied expression distributions in different samples. *Asterisks* indicate significance levels at *p < 0.05, **p < 0.01, and ***p < 0.001. (*b*) Percentages of tumor-infiltrating immune cells in each sample. *Different colors* depict the different types of immunological cells. The *abscissa* denotes the sample, whereas the *ordinate* denotes the percentage of immune cells in a single sample. **(H)** Immunomodulators, chemokines, and receptors associated with *FCN2* in liver hepatocellular carcinoma (LIHC). **(B)** CCL14. **(C)** CCL16. **(D)** CCL23. **(E)** TNFSF4. **(F)** CD276. **(G)** TNFRSF4. **(H)** KDR. ****p < 0.0001.

*FCN2* was found to be favorably linked to the majority of immunomodulators, chemokines, and other factors, as well as the chemokine receptors in LIHC and diffuse large B-cell lymphoma (DLBCL), and negatively correlated with most of the immunomodulators, chemokines, and chemokine receptors in THYM and TGCT. *FCN2* was positively correlated with most major histocompatibility complexes (MHCs) in LIHC, mesothelioma (MESO), and acute myeloid leukemia (LAML) and negatively correlated with most MHCs in TGCT (**Figure 10A**).

Analysis using the TISIDB database showed that three chemokines, CCL14 (*ρ* = 0.432, *p* < 2.2E−16), CCL16 (*ρ* = 0.232, *p* = 6.21E−06), and CCL23 (*ρ* = 0.402, *p* = 2.2E−16), were positively correlated with the expression of *FCN2*. The immunosuppressant kinase insert domain receptor (KDR) (*ρ* = 0.294, *p* = 8.34E−09) was also positively linked to the expression of *FCN2*. However, *FCN2* expression was found to be inversely correlated with the expression of four chemokine receptors, namely, CD276 (*ρ* = −0.239, *p* = 3.19E−06), TNFRS4 (*ρ* = −0.204, *p* = 7.72E−05), CD276 (*ρ* = −0.239, *p* = 3.19E−06), and TNFSF4 (*ρ* = 0.211, *p* = 4.27E−05) (**Figures 10B–H**).

**Figure 11:**
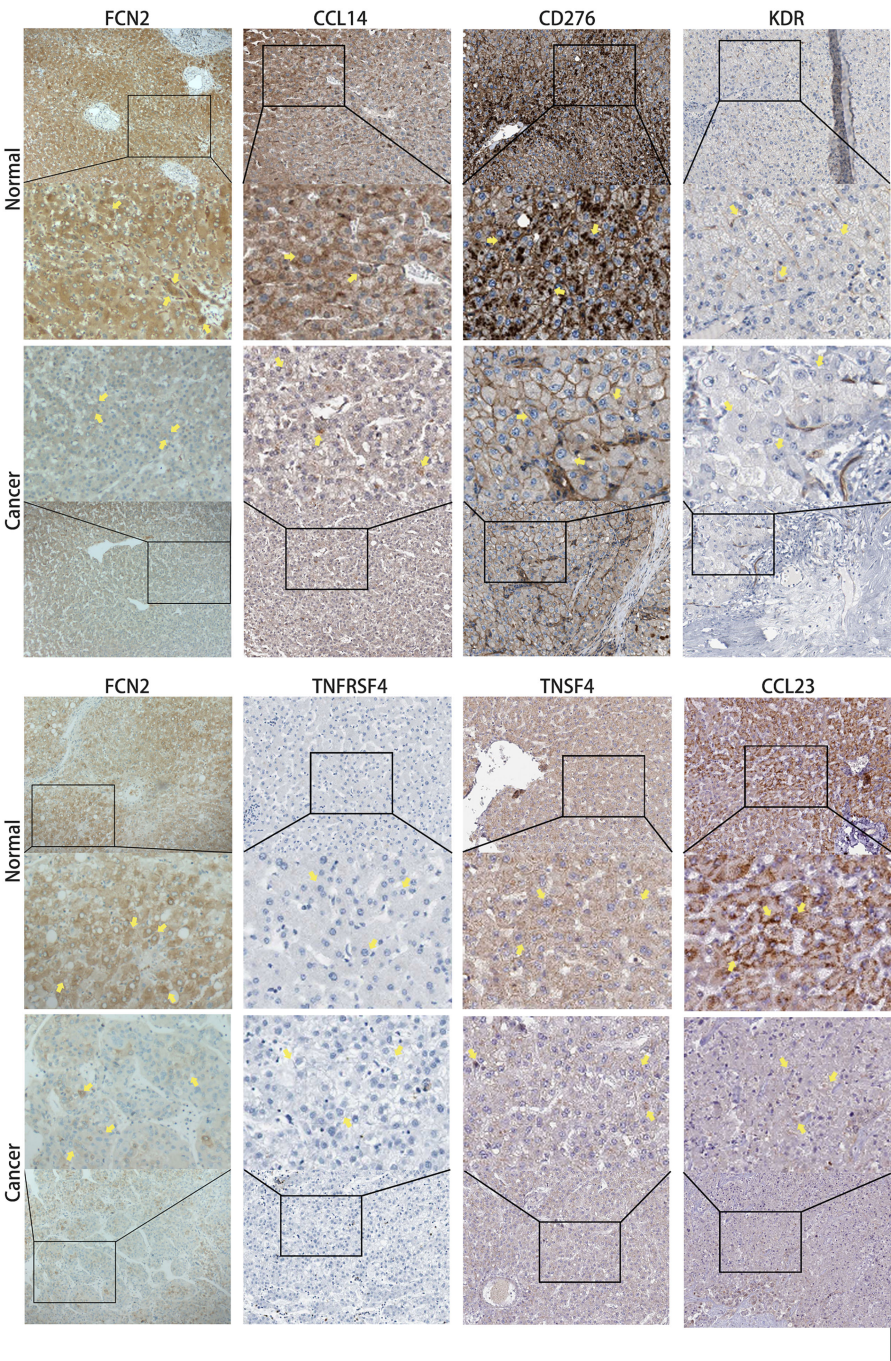
Immunohistochemical comparison of *FCN2* and the molecules with strong correlations [based on the Human Protein Atlas (HPA)].

The HPA database confirmed that the expression levels of the immune-related markers CCL14, CCL23, KDR, CD276, TNFSF4, and TNFRSF4 in liver cancer are closely linked to *FCN2*. Apart from the lack of significant differences in the levels of positive rates, the immunohistochemistry results of the remaining molecules revealed that the positive rates in normal liver tissues were greater than the high specificity in liver cancer tissues, meaning that the expressions of these genes were lower in liver cancer tissues than those in normal liver tissues. The evidence shown in **Figure 11** backs this up. These findings show that the expression of *FCN2* is TME-specific, implying that *FCN2* could be a potential immunotherapy target in the treatment of liver cancer.

## Discussion

It has been demonstrated that gene therapy can be used to treat cancer, specifically liver cancer. It examines differences in the gene expression levels in liver cancer, determines which genes are expressed differently, and then intervenes in their expression. The mRNA expression levels of *FCN2* in liver cancer tissues were lower than those in normal liver tissues, showing that *FCN2* may play the role of a tumor marker in the occurrence and progression of liver cancer. The *FCN2* gene is a polymorphic gene with functional polymorphisms that control its expression and function. It may have a pathophysiological role in innate immunity (14).

Using the Oncomine database, we determined that the expression of *FCN2* in LIHC tissue was inferior to that in normal liver tissue. The expression of *FCN2* mRNA in liver cancer tissues was similarly shown to be lower than that in normal liver tissues. We further analyzed the Clinical Proteomic Tumor Analysis Consortium (CPTAC) database and discovered that the FCN2 protein expression in liver cancer tissues was inferior to that in normal liver tissues. The *FCN2* gene was analyzed using the UALCAN online analysis tool, which gave the same results as above. According to studies, the serum levels of *FCN2* in patients with tumors are substantially lower than those in healthy individuals. Thus, *FCN2* has an antitumor effect (15). This finding further supports our assumption. We used the Oncomine database to examine the expression of *FCN2* mRNA in malignant tumors and normal clinical tissues in order to determine whether *FCN2* has a specific expression in liver cancer. Downregulation of the gene expression of *FCN2* was found in leukemia and liver cancer. RNAseq (transcriptome gene sequencing technology) and Affymetrix were used for further verification, and the results showed that tumor type was the main factor affecting the transcription level of *FCN2*.

The results obtained by TCGA and the other online databases, together with the high-throughput RNAseq results of the bioinformatics analysis verified by experiments, showed that the expression of *FCN2* in HCC tissues is lower than that in normal liver tissues. This study found that *FCN2* appears to play a role in the development and progression of liver cancer. Furthermore, receiver operating characteristic (ROC) analysis revealed that *FCN2* has an area under the curve (AUC) of 0.840 for the diagnosis of HCC, implying that it could be a potential diagnostic biomarker. The relationship between *FCN2* expression and the clinicopathological features was further studied based on this concept. According to the univariate analysis, the low protein expression of *FCN2* is substantially linked to height (*p* = 0.024) and the AFP level (*p* = 0.004). To determine the link between the *FCN2* gene and liver cancer, we used the R package to analyze the *FCN2* gene expression in multiple indicators. The expression of *FCN2* was shown to differ among the AFP groups.

Previous studies have supported the hypothesis that genetic mutations can influence the development of liver cancer (16). To explore whether *FCN2* has a similar mechanism, the cBioPortal database was utilized to examine *FCN2* mutations in liver cancer based on the sequencing data from patients in the TCGA database. The most common *FCN2* mutation in HCC is amplification. However, the Kaplan–Meier plot showed that the *FCN2* gene mutation had no effect on the prognosis of HCC.

In the analysis of the PPI network diagram of 10 genes and the *FCN2* gene using the STRING database, GO, and KEGG signal pathways, we discovered that the *FCN2* gene may play a key role in *S. aureus* infection, complement coagulation cascade, and lectin complement pathway.

Numerous studies have found that different types of immune cells infiltrate tumors in large numbers and that their distribution, tissue location, and cell type are all linked to tumor development and survival (17). We found that the expression levels of *FCN2* are abnormally low in HCC. Based on our findings, *FCN2* may play a role in modulating the tumor immune response. We started by examining the *FCN2* expression in pan-cancer. We found that it was positively correlated with most immune cells in LAML, skin cutaneous melanoma (SKCM), bladder urothelial carcinoma (BLCA), LUAD, pancreatic adenocarcinoma (PAAD), pheochromocytoma and paraganglioma (PCPG), KIRP, and lung squamous cell carcinoma (LUSC) and was also positively correlated with activated CD4^+^ T cells in LIHC. Eosinophils and CD56^bright^ NK cells were found to be negatively and favorably linked, respectively, to activated DCs. We then looked at the relationship between *FCN2* expression and immune infiltration in HCC and found that the expression of *FCN2* was positively linked to the number of neutrophils, eosinophils, NK cells, Tcm, and DCs infiltrating the tumor. The expression level of *FCN2* had a negative correlation with Th2 cell infiltration. Neutrophils are frequently dominant and are linked to immunological escape from tumors (18). Consequently, these findings imply that *FCN2* may suppress the HCC tumor immune response by favorably regulating neutrophils (19, 20), eosinophils (21), NK cells (22–24), Tcm (25), and DCs (26–28) and negatively regulating Th2 (29–31) in tumors.

Blood arteries, immune cells, fibroblasts, bone marrow-derived inflammatory cells, various signaling molecules, and the extracellular matrix (ECM) were all present in the close vicinity of tumor cells. The TME facilitates complex interactions between tumor cells and stromal cells (32). One of the immunological characteristics of the TME is the expression of immunomodulatory chemicals and inhibitory immune checkpoints. The *FCN2*-related pan-cancer analyses were designed to describe the immune effects of *FCN2* and are critical in identifying cancer types that may benefit from *FCN2*-related immunotherapy. In LIHC and DLBCL, *FCN2* was shown to be positively correlated with the majority of immunomodulators, chemokines, and chemokine receptors, but was negatively correlated with the majority of these same molecules in THYN and TGCT. *FCN2* was positively correlated with most MHCs in LIHC, MESO, and LAML and negatively correlated with most MHCs in TGCT. In addition, we found that three chemokines—CCL14, CCL16, and CCL23—and the immunosuppressant KDR were positively correlated with the expression of *FCN2*, while three immunostimulants—CD276, TNFRSF4, and TNFSF4—were negatively correlated with its expression. Immunohistochemical analysis revealed that the positive rates of CCL14 (33, 34), CCL23 (35, 36), CD276 (37, 38), and TNFSF4 (39) were significantly different in normal liver and liver cancer tissues.

Chemokines have been found to impact the biological activity of inflammatory cells, implying that the TME can operate as inflammatory cell regulators (40). The capacity of cancer tissues to spread metastatically could be linked to the metastatic potential of liver cancer (41). Chemokines and their receptors are considered to have a role in HCC development, invasion, and metastasis (42). CCL14, for example, may prevent the Wnt/catenin pathway from engaging, reducing HCC cell growth, and increasing apoptosis (34). CCL23 has been found to be underexpressed in hepatoma cells, and this could lead to CCL23 deletion and reduced CCL23 inhibition *via* the ESR1/CCL23/CCR1/AKT regulatory axis in liver cancer progression (35).

Studies on the clinical application of *FCN2* in the treatment of liver cancer discovered two kinds of ficolin in mice, namely, *Fcna* and *Fcnb*. The lectin pathway is activated by both mouse *Fcna* and human *FCN2*, which are produced in the liver and present in the blood (43). The Fcnb levels are very low, whereas those of *Fcna* and the structure in host plasma are identical to those seen in mantle cell lymphoma (monoclonal B-cell lymphocytosis, MBL) (15, 44, 45). This shows that the mouse *Fcna* can be used instead of the human *FCN2* for experimental analysis to obtain more relevant indicators of *FCN2*.

Finally, we obtained some clinical samples for immunohistochemical dissection for the evaluation and validation of the accuracy of our results. *FCN2* expression was shown to be lower in liver cancer tissues than in healthy liver tissues, which is consistent with our other findings.

Therefore, we propose exploring gene therapy targeting *FCN2*. Gene therapy may be widely used clinically in the future (46). Molecular tools can also be used to target gene therapy in the liver (47). Based on this, this paper discusses the influence of the *FCN2* gene on liver cancer and expects to expand the research in the future, more fully analyze the association between this gene and liver cancer, and apply gene therapy to better treat liver cancer.

## Conclusions

Our experimental evidence and the meaningful correlation with AFP support the finding that *FCN2* is underexpressed in HCC and that it has prognostic value in this cancer type (4, 7). *FCN2* may be implicated in *S. aureus* infection, complement coagulation, and the lectin complement pathway in terms of function. At the immune level, *FCN2* may suppress the tumor immune response of HCC by upregulating the entry of neutrophils, eosinophils, NK cells, Tcm, and DCs and downregulating the entry of Th2 into tumors. *FCN2* also interacts with CCL14, CCL23, KDR, CD276, TNFSF4, and TNFRSF4. Because these immune-related associations are so significant, *FCN2* may be developed as an immune checkpoint inhibitor for liver cancer. However, there is still potential for improvement in our research because the precise involvement of the *FCN2* gene in the onset and progression of liver cancer, as well as its mechanism of impact, remains unclear, and many hypotheses must be confirmed by clinical data. It is hoped that by analyzing the mechanism and characteristics of *FCN2* in the development of HCC, this study can provide useful information for the future of liver cancer.

## Data availability statement

Publicly available datasets were analyzed in this study. These data can be found here: National Center for Biotechnology Information (NCBI) Gene Expression Omnibus (GEO), https://www.ncbi.nlm.nih.gov/geo/, GSE45267, GSE87630, and GSE54236.

## Ethics statement

Written informed consent was obtained from the individual(s), and minor(s)’ legal guardian/next of kin, for the publication of any potentially identifiable images or data included in this article.

## Author contributions

XL contributed to the experimental idea and guidance. L-tW designed the experiments. Z-yC and X-lW collected the data. S-lJ and Q-lZ participated in the data analysis and interpretation. L-tW, Z-yC, X-lW, S-lJ, and Q-lZ drafted the manuscript. LL did an immunohistochemical test. L-tW revised the manuscript. All authors contributed to the article and approved the submitted version.

## Funding

The Natural Science Foundation of Guangxi Province (grant no. 2017GXNSFAA198063) and the Guangxi Medical University’s Basic Medical Science and Technology Innovation Training Fund Project (grant no. GXMUBMSTCF-G15) sponsored this research.

## Acknowledgments

The authors express their gratitude to the members for their contributions to this effort.

## Conflict of interest

The authors declare that the research was conducted in the absence of any commercial or financial relationships that could be construed as a potential conflict of interest.

## Publisher’s note

All claims expressed in this article are solely those of the authors and do not necessarily represent those of their affiliated organizations, or those of the publisher, the editors and the reviewers. Any product that may be evaluated in this article, or claim that may be made by its manufacturer, is not guaranteed or endorsed by the publisher.

## Supplementary material

Attachment 1 FCN2mRNA expression in different cells. **(A)**,FCN2 mRNA expression in different cell lines was analyzed based on RNAseq (transcriptome gene sequencing technology). **(B)**,Analysis of FCN2 mRNA expression in different cell lines based on Affy.

## Abbreviations

BP, biological processes; CC, cell components; DAVID, the Database for Annotation, Visualization and Integrated Discovery; DEGs, differentially expressed genes; GEO, Gene Expression Omnibus; GEPIA, Gene Expression Profiling Interactive Analysis; GO, Gene Ontology; GTEX, Genotype-Tissue Expression; HCC, hepatocellular carcinoma; KEGG, Kyoto Encyclopedia of Genes and Genomes; LASSO, least absolute shrinkage and selection operator; MF, molecular functions; TCGA, The Cancer Genome Atlas.
