# Supplementary figures and images for: Ficolin-2: A potential immune-related therapeutic target with low expression in liver cancer
Source: Front Oncol. 2022 Nov 8;12:987481. doi: 10.3389/fonc.2022.987481 (PMC9679423; doi:10.3389/fonc.2022.987481)

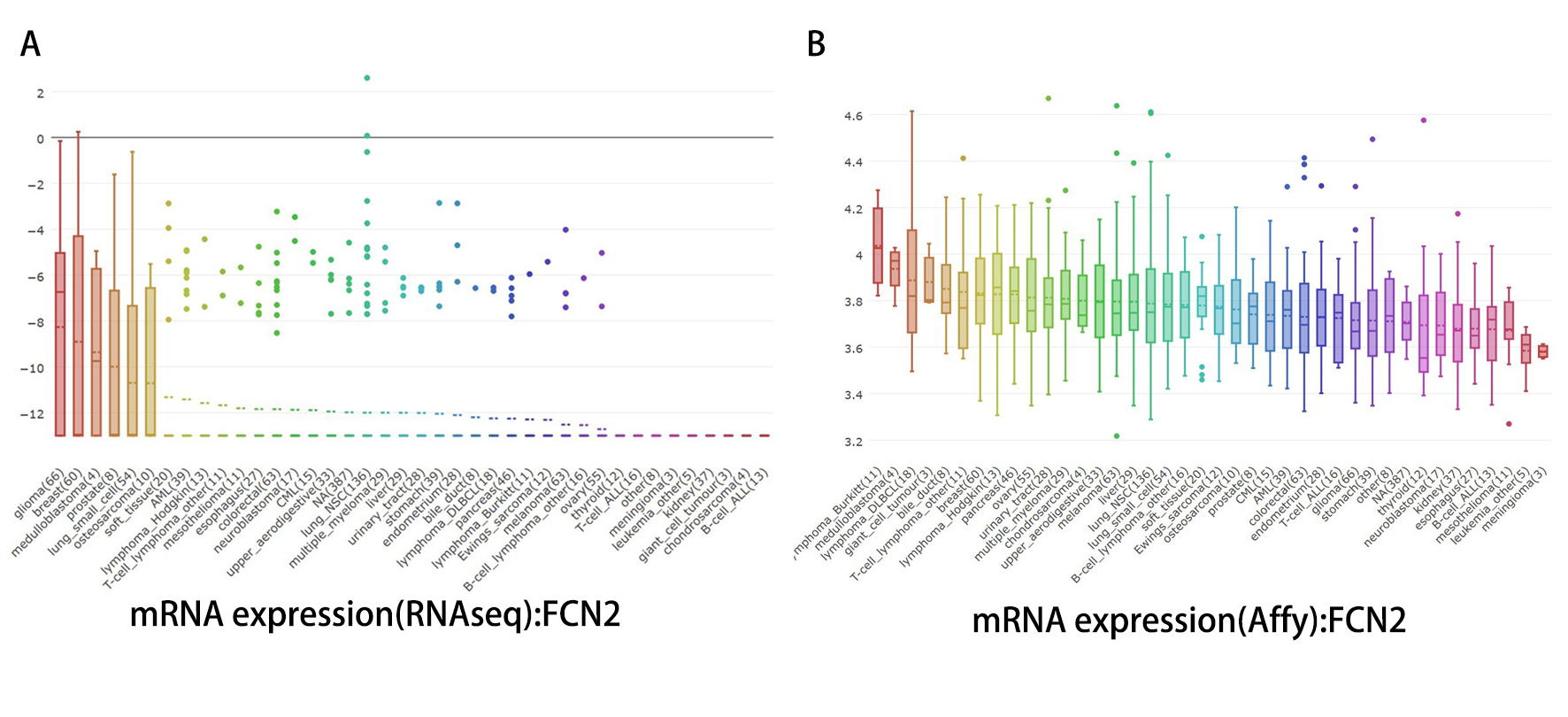

Supplement: Attachment 1 — FCN2mRNA expression in different cells. (A),FCN2 mRNA expression in different cell lines was analyzed based on RNAseq (transcriptome gene sequencing technology). (B),Analysis of FCN2 mRNA expression in different cell lines based on Affy. [file Image_1.jpeg]
